# Seclusion as a coercive measure in suicidality – daily routine or exception?

**DOI:** 10.1186/s12888-022-04076-x

**Published:** 2022-06-25

**Authors:** Natalija Gavrilovic Haustein, Maurice Freudiger, Anna Hunziker, Urs Hepp, Lena Jellestad, Roland von Känel, Niklaus Stulz

**Affiliations:** 1 Integrated Psychiatric Services Winterthur – Zurcher Unterland, P.O. Box 144, CH-8408 Winterthur, Switzerland; 2 Meilener Institute Zurich, Stockerstrasse 45, Zurich, CH-8002 Switzerland; 3 Department of Consultation-Liaison Psychiatry and Psychosomatic Medicine, University Hospital Zurich, University of Zurich, Zurich, Switzerland

**Keywords:** Coercive measures, Seclusion, Suicidality

## Abstract

**Background:**

Coercive measures continue to be an important topic in psychiatry. However, there is no proof of the effectiveness of the use of coercive measures, especially with suicidal people. For many years, attempts have been made to replace such measures with alternative noncoercive intervention options. This paper aims to clarify the situation of coercive measures, more precisely seclusions, in a general psychiatric hospital in Switzerland. It focuses on compulsory measures in patients with suicidal tendencies.

**Method:**

In this single-centre retrospective cohort study, we used routinely collected medical data and performed qualitative analyses of medical histories to examine whether alternative measures to seclusion had been offered and/or provided to patients who had been secluded solely because of suicidality. Patients were aged 18–65 years and had received inpatient treatment at one of five adult acute care units at a general psychiatric hospital in Switzerland between September 2016 and December 2019.

**Results:**

There were 5,935 inpatient treatment cases during the study period. Suicidality was rated as “acute” or “very high” at least once during the hospitalization in 219 (3.7%) cases. Of these, 60 were excluded from further analyses as they involved seclusion, but suicidality was not the exclusive indication for this measure. Coercive seclusion was imposed exclusively due to suicidality in 53 (33.3%) of the remaining 159 cases, whereas 106 (66.7%) cases were not secluded. The rates of seclusion among suicidal patients varied considerably between the hospital wards (13.0% to 55.3%). Suicidal patients with non-Swiss residence status and/or lacking language skills were particularly prone to be secluded. Additionally, alternative interventions were offered and provided significantly more frequently in the nonsecluded patients.

**Conclusions:**

To avoid seclusion due to suicidal tendencies, it is necessary to have a general attitude of avoiding coercive measures at all costs. It is also important for qualified staff to be able to deal with challenging sociodemographic characteristics of patients such as foreign-language, which may require translators and intercultural interpreters.

## Background

Coercive measures in psychiatry represent a massive infringement on the patient's privacy and right to self-determination, leading to inherent ethical dilemmas and conflicts between self-determination and the patient's well-being [1]. Current psychiatric care aims to limit coercive interventions whenever possible [2]. However, clinical reality shows that coercive measures continue to be applied in psychiatric hospitals under certain circumstances, e.g., when the patient exhibits harm to self or others. However, coercive measures have no explicit recommendation, nor has research been able to demonstrate a clear advantage for them [3]. In the medical context, coercive measures include all measures that are administered against the patient's self-determined will or with the patient’s resistance [4].

Acute or admission wards of psychiatric hospitals provide care for patients in acute crises. Admission may be voluntary or, if imperative, against a patient's will, subject to the respective Swiss legal framework [4].

Systematic research on coercive treatment is scarce [5] due to limitations arising from ethical and legal arguments [6]. Both internationally and nationally, there are differences in the frequency and types of coercive measures applied [7], which suggests different cultural approaches to coercive measures across health care settings.

Coercive measures are applied in the event of a patient exhibiting harm to self or others. The latter situation mostly arises from acute states of agitation and aggression. Harm to self typically results from acute suicidality or severe self-injurious behaviour. Suffering from a mental illness is a major risk factor for the development of suicidality [8, 9]. Accordingly, suicidal patients are common in psychiatric or psychotherapeutic care [10]. Suicide prevention and therapeutic support for people in suicidal crises are therefore a core mission of psychiatry [11].

The aim of this study was to examine the use of seclusion as a coercive measure for suicidality in a general psychiatric hospital. The objective was to explore whether and how often seclusion was applied to suicidal patients in clinical practice, whether and which alternative interventions were offered or provided in advance, and thus whether seclusion truly represented a "remedy of last resort". Moreover, we aimed to assess whether there were differences in sociodemographic and clinical characteristics between suicidal patients who were and were not secluded.

The present study aimed to provide insights into the use of coercive measures in everyday clinical practice. This should help to identify which alternative noncoercive intervention options may be taught to and implemented by staff in order to reduce coercive measures in suicidal patients.

## Methods

### Study design and participants

This single-centre retrospective cohort study was based on routinely collected medical data. Qualitative analyses of medical histories were performed to identify whether patients had been secluded solely because of suicidality and whether alternative measures to seclusion had been offered and/or provided to suicidal patients. Participants comprised patients aged 18–65 years who received inpatient treatment at one of five adult acute care units at a psychiatric hospital in Switzerland between September 2016 and December 2019. We included patients who had been rated as having "acute" or "very high" suicidality in the standard diagnostic assessment of suicidality (see below) at least once during the course of their inpatient treatment. Patients who had been referred from penal institutions were excluded. Data collection began in September 2016 because indications for coercive measures were systematically recorded from this moment.

### Measures

#### Suicide risk assessment

Suicide risk assessment was repeatedly performed during the course of treatment through standardized clinical assessments (CAs) and/or the German version of the Nurses’ Global Assessment of Suicide Risk (NGASR) [12], a systematic and reliable 15-item measure of evidence-based risk factors for suicide [13]. In the NGASR, the answer options yes/no are used to assess the presence of the risk factors. Each risk factor is weighted by assigning a point value. Five particularly important risk factors (e.g., prior suicide attempt) are assigned 3 points, and the others (e.g., recent stressful life event) are assigned 1 point. The total score ranges from 0–25 points. Cutcliffe and Barker recommended the following categorization of risk levels: 0–5 points = low risk; 6–8 points = moderate risk; 9–11 points = high risk; 12 and more points = very high risk [13]. The results of the CAs were documented as "none", "elevated" or "acute". The instruments were rated by either therapists (CA) or interprofessionally by therapists and nurses (NGASR) at intake, at discharge and whenever there was a perceived change in the patient's condition.

#### Coercive treatment

Since the standardized documentation only indicated harm to others and/or self-harm as reasons for coercive measures, cases of patients being secluded solely for suicidality had to be identified by reviewing medical records. Cases with coercive measures due to self-harm and a rating of "acute" or "very high" suicidality were preselected and then reviewed by trained raters based on medical records. In a subsequent qualitative data analysis, cases of other types of self-harm were excluded to identify suicidality as the sole indicative criterion. In cases with multiple seclusions solely due to acute suicidality, we considered the first episode only. We further assessed whether patients were offered or provided with one or more alternative intervention to seclusion, as recommended by the Academic Society of Psychiatric Nursing of the Swiss Association for Nursing Science [14] and the German Society for Psychiatry, Psychotherapy and Neurology [1]: “one consultation", "multiple consultations", "stay within sight of the nursing staff", "active surveillance" (nurses are permanently nearby and keep an eye on the patient), "passive surveillance" (patient reports back to nurses in the case of need), "medication without coercion" (the patient is willing to take the medication) or "one-to-one” intensive care. Raters were also instructed to document alternative interventions not included in the measures above. Two independent raters reviewed the seven potential alternative measures in nine cases and showed good interrater reliability (percentage of agreement = 85.7%).

### Statistical analysis

Sociodemographic and clinical characteristics as well as alternative interventions for seclusion were compared between secluded and nonsecluded patients using Fisher's exact, χ^2^, Mann–Whitney or independent-samples t tests as appropriate.

## Results

**Fig. 1:**
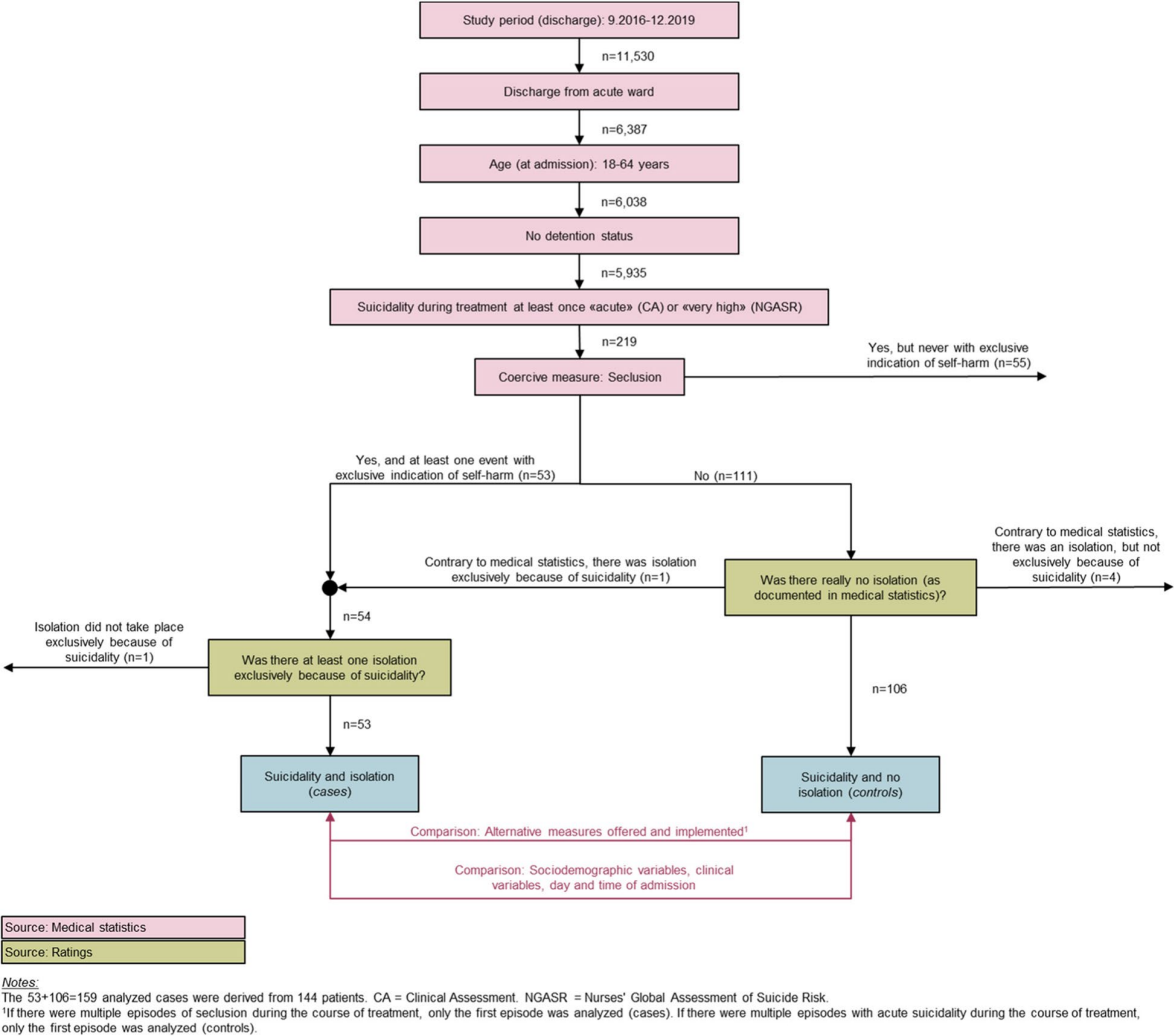
Flowchart

Between September 1, 2016, and December 31, 2019, a total of 5,935 inpatient treatment episodes (cases) of patients aged 18–64 years were completed at the five acute wards (Fig. 1). Suicidality was rated as being "acute" (CA) and/or "very high" (NGASR) at least once in 219 (3.7%) of these cases (Fig. 1). Sixty of these 219 cases were excluded from further analyses, as suicidality was not the exclusive indication for seclusion. In 53 (33.3%) of the remaining cases, coercive seclusion was imposed exclusively due to acute or very high suicidal tendencies, while in 106 (66.7%) cases with acute or very high suicidality, seclusion was not imposed.

Table 1 displays the sociodemographic and clinical characteristics comparing secluded and nonsecluded suicidal patients.

**Table 1:** Sociodemographic and clinical characteristics of secluded and nonsecluded suicidal patients

		Secluded patients (*n* = 53) *n* (%)/*M (SD)*	Nonsecluded patients (*n* = 106) *n* (%)/*M (SD)*	*p value*
Age		35.5 (13.9)	38.3 (13.6)	.164
Sex	Female	26 (49.1%)	52 (49.1%)	.999
Main diagnosis at discharge^a^	F1	6 (11.3%)	9 (8.6%)	.298
	F2	7 (13.2%)	6 (5.7%)	
	F3	17 (32.1%)	51 (48.6%)	
	F4	12 (22.6%)	22 (21.0%)	
	F6	9 (17.0%)	15 (14.3%)	
	Other	2 (3.8%)	2 (1.9%)	
Number of secondary diagnoses		1.36 (1.33)	1.12 (1.16)	.318
Secondary diagnosis of a personality disorder (F6)		2 (3.8%)	16 (15.1%)	.034
Compulsory admission		41 (77.4%)	47 (44.3%)	< .001
Communication (language skills)	Not possible	11 (20.8%)	6 (5.7%)	.003
	Difficult	6 (11.3%)	5 (4.7%)	
	Possible	36 (67.9%)	95(89.6%)	
Intoxication at admission		16 (30.2%)	24 (22.6%)	.301
Admission by police		17 (32.1%)	13 (12.3%)	.003
Admission during office hours		13 (24.5%)	24 (22.6%)	.791
Temporary retention^b^		11 (20.8%)	2 (1.9%)	< .001
Residence status^c^	Swiss	29 (54.7%)	74 (71.8%)	.001
	Foreigner	3 (5.7%)	17 (16.5%)	
	Asylum status	11 (20.8%)	6 (5.8%)	
	Other	10 (18.9%)	6 (5.8%)	
Residence prior to admission	At home	20 (41,7%)	68 (66.7%)	.003
	Residential home/hospital	16 (33.3%)	28 (27.5%)	
	Homeless	3 (6.2%)	2 (2.0%)	
	Other	8 (16.7%)	3 (2.9%)	

Notes: Group comparisons were performed using Pearson’s chi^2^ tests, Fisher's exact tests, Mann–Whitney U tests or t tests

^a^Diagnoses according to the German version of the International Statistical Classification of Diseases and Related Health Problems (ICD-10) of the World Health Organization (WHO). Information on the main diagnosis was missing for one patient who was not secluded (0.9%)

^b^According to article 427 of the ZGB (Swiss civil code) of the SGB (social code), persons admitted voluntarily may be retained in the hospital against their will for a maximum of 72 h if there is an acute danger to themselves or others

^c^Information on residence status was missing for three patients who were not secluded (2.8%)

Age, sex and primary psychiatric diagnoses did not differ between groups. In both groups, affective (ICD-10: F3) and stress-related (F4) diagnoses were most prevalent. Patients who had been admitted compulsorily or who were admitted by police were significantly more likely to be secluded than patients who sought medical care voluntarily. The use of seclusion was more frequent in patients with poor knowledge of German and in asylum seekers (refugees who apply for asylum in Switzerland for political reasons). Time of admission did not correlate with the use of seclusion as a coercive measure.

All patients who were not secluded received at least one alternative measure to avoid seclusion (Table 2). In contrast, in 7 (13.2%) of the cases of seclusion, no alternative measure was offered to the patients, and another 6 (11.3%) of the patients refused the alternative measures offered to them. As shown in Table 2, “one conversation” was the most frequent alternative measure in both groups, and it was more frequent in patients who were not subsequently secluded than in those who were secluded (98.1% vs. 71.7%, *p* < 0.001). “Active surveillance” (nurses nearby) was also applied significantly more often in patients who were not secluded (43.4% vs. 0%, *p* < 0.001). Passive monitoring was rarely offered or implemented in either group (5.6% vs. 1.9%; *p* = 0.78). Patients who were subsequently secluded were significantly less likely to be given noncoercive medications, and when offered, they were more likely to refuse such medication. One-to-one intensive care was rarely offered or provided in either group (3.8% vs. 2.9%, *p* = 0.99). Further documented alternative measures included offering a cigarette, offering to contact relatives or offering food or drinks. They were primarily applied in the nonsecluded group. The rates of seclusion among suicidal patients varied considerably between units, from 13.0% to 55.3% (*p* < 0.001). Coercive seclusions due to suicidality lasted on average M = 33.1 h (SD = 30.0, Range: 2.8–135.5).

**Table 2:** Frequencies of specific measures in secluded and nonsecluded suicidal patients

Intervention		Secluded patients (*n* = 53) *n* (%)	Nonsecluded patients (*n* = 106) *n* (%)	*p value*
One conversation	Applied Offered, but refused Not offered	38 (71.7%) 6 (11.3%) 9 (17.0%)	104 (98.1%) 1 (0.9%) 1 (0.9%)	< .001
Multiple conversations	Applied Offered, but refused Not offered	2 (3.8%) 2 (3.8%) 49 (92.5%)	32 (30.2%) 1 (0.9%) 73 (68.9%)	< .001
In visual range of nursing staff	Applied Offered, but refused Not offered	1 (1.9%) 1 (1.9%) 51 (96.2%)	4 (3.8%) 0 (0.0%) 102 (96.2%)	.557
Active surveillance^b^	Applied Offered, but refused Not offered	0 (0.0%) 0 (0.0%) 53 (100%)	46 (43.4%) 0 (0.0%) 60 (56.6%)	< .001
Passive surveillance^c^	Applied Offered, but refused Not offered	1 (1.9%) 0 (0.0%) 52 (98.1%)	5 (4.7%) 1 (0.9%) 100 (94.3%)	.776
Medication (without coercion)	Applied Offered, but refused Not offered	13 (24.5%) 8 (15.1%) 32 (60.4%)	66 (62.3%) 5 (4.7%) 35 (33.0%)	< .001
One-to-one intensive care^a^	Applied Offered, but refused Not offered	2 (3.8%) 0 (0.0%) 51 (96.2%)	3 (2.9%) 0 (0.0%) 102 (97.1%)	.999
Open seclusion room/voluntarily seclusion	Applied Offered, but refused Not offered	0 (0.0%) 0 (0.0%) 53 (100.0%)	9 (8.5%) 0 (0.0%) 97 (91.5%)	.030
Smoking a cigarette	Applied Offered, but refused Not offered	2 (3.8%) 0 (0.0%) 51 (96.2%)	2 (1.9%) 0 (0.0%) 104 (98.1%)	.601
Contact family/relatives/reference person	Applied Offered, but refused Not offered	0 (0.0%) 0 (0.0%) 53 (100.0%)	11 (10.4%) 0 (0.0%) 95 (89.6%)	.016
Eat/drink something	Applied Offered, but refused Not offered	0 (0.0%) 0 (0.0%) 53 (100.0%)	7 (6.6%) 1 (0.9%) 98 (92.5%)	.096
Other intervention^d^	Applied Offered, but refused Not offered	0 (0.0%) 0 (0.0%) 53 (100.0%)	9 (8.5%) 0 (0.0%) 97 (91.5%)	.030
At least one of the aforementioned interventions	Applied Offered, but refused Not offered	40 (75.5%) 6 (11.3%) 7 (13.2%)	106 (100.0%) 0 (0.0%) 0 (0.0%)	< .001

Notes: Group comparisons were performed using Pearson’s chi^2^ tests, Fisher's exact tests, Mann–Whitney U tests or t tests

^a^Information was missing value for one nonsecluded patient

^b^Nurses proactively observe the patient

^c^Patients are instructed to report to the nursing staff immediately in the case of crisis

^d^Developing skills, going to sleep, receiving an infusion, signing a nonsuicide contract, etc.

## Discussion

Although most hospitals advocate avoiding coercive measures in general and seclusion of suicidal patients in particular, our results showed that seclusion was in fact applied in one-third (33.3%) of the 159 inpatient treatments for patients with acute or very high suicidality. These patients were secluded for a mean duration of M = 33.1 h despite written in-house guidelines which recommend avoidance of seclusion for suicidal patients whenever possible. During seclusions, patients were contacted by staff according to prior and mutual agreement. However, a contact happened at least every 30 min, as there was no video recording to monitor patients in seclusion rooms. Additional measures to seclusion such as medication or monitoring of somatic parameters (e.g. oxygenation) were tailored to the patients' individual needs.

Two categories of coercive measures can be distinguished: measures to restrict freedom as security measures and medical measures that serve diagnostic or therapeutic purposes [15]. In Switzerland, the use of coercive measures is strictly regulated by law: they may only be applied in urgent and life-threatening circumstances to save lives or avert serious harm [16]. The chosen measures must be appropriate [17] and provided in a location that is suitable both in terms of infrastructure and personnel [4]. Coercive measures should always be a “last resort” [18]. There are various forms of coercive measures: seclusion, mechanical restraint such as fixation (fixing the patient with belts on the bed), and coercive medication (oral or intramuscular). If previously admitted patients were returning to our hospital and had a "preferred treatment" form which specified their preferences with regard to medication or use of coercive interventions, this was taken into account as a matter of course. However, the treatment of acute suicidal tendencies implies a high density of care [19], which does not correspond to a separation from others in a shielded room.

A study assessed the use of coercive measures in 24 psychiatric admission units of 12 hospitals in the German-speaking part of Switzerland. Coercive measures were applied in 13% of treatment episodes. Seclusion was used most frequently (31%), followed by seclusion combined with medication (25%), and fixation (9%) [20]. In the Europe-wide EUNOMIA study, coercive measures such as coercive medication, fixation, or seclusion were applied in 38% of approximately 2000 compulsorily admitted patients. Whereas fixation was the most prevalent coercive measure in Germany, coercive medication was most frequently applied across Europe. Seclusion was only permitted in six of the countries studied [7]. Compared to Germany, the proportion of seclusion and fixations in Switzerland is higher [21]. A Swiss study comparing psychiatric hospitals in the canton of Zurich also pointed to differences within the same region [22]. In that study, 24.8% of hospitalizations were involuntarily and seclusion and coercive medication were applied in 6.4% and 4.2% of cases, respectively. In the 2019 report of the National Association for Quality Development of Hospitals and Clinics (ANQ), 32 of 37 acute and primary care hospitals reported using measures that restrict patients’ freedom. Across all adult psychiatric facilities, the proportion of treatment cases with at least one coercive measure was 7.7% [23].

One way to avoid coercive measures is to create a general attitude that is obligatory for all professionals and to provide and apply alternative measures. The present study demonstrated that consultations and active monitoring by nurses were provided more frequently for nonsecluded patients than for secluded patients. Further alternative interventions, such as offering a cigarette or something to eat, or involving relatives, were also provided significantly more frequently to nonsecluded patients. Intensive care emphasizes and supports a relationship-based and trusting approach with simultaneous protection and safety-providing interventions [24, 25]. Intensive care interventions should allow for the formation of a good therapeutic relationship [26]. Operational and organizational conditions are required for providing cost-intensive intensive care in clinics [14]. Suicide risk assessment with adapted interventions has proven to be an effective means of standardized suicide treatment [27]. Internal clinical standards can help regulate clinical processes and clarify courses of action. Such standards also necessitate a general attitude of noncoercive psychiatry.

As aforementioned, alternative measures were less frequently provided to secluded patients, and they were more often rejected by secluded patients. One consideration might therefore involve the distribution of resources – are enough staff available to devote extended time to the patient? An initial rejection by the patient should not be immediately followed by coercive action; instead, several attempts for contact should be undertaken. The objective when treating suicidal patients in acute psychiatric wards is to provide "first aid in mental distress". In this situation, any engagement with suicidal people may already be regarded as the beginning of a psychotherapeutic intervention [28]. In particular, establishing stable rapport with patients is considered essential and the most important intervention for suicidal crises [29]. Suicidal patients need the presence of others; therefore, seclusion should be avoided whenever possible and must be applied on the basis of strict indication criteria only. Close monitoring is always preferable, as separation leaves the suicidal patient to his or her own devices [9]. In cases where seclusion is applied shortly after admission, establishing a therapeutic relationship is hardly possible. Questions of proportionality also arise in cases where alternative measures are not provided. Unfortunately, the reasons for not providing alternative measures were not apparent in the qualitative survey of the medical records. Therefore, one can only speculate about the reasons.

Given the focus on suicidality in our study, it is not surprising that ICD-10 diagnoses of affective (F3) and anxiety and stress-related disorders (F4) were most prevalent in both groups. Contrary to our expectations, diagnoses of schizophrenia (F2) and substance abuse (F1) were not statistically significantly more prevalent in secluded patients than in nonsecluded patients. This contradicts the findings of a study among German psychiatric hospitals, which revealed a higher incidence of coercive measures for these two diagnostic groups. However, that previous study also included coercive measures which had been imposed due to potential harm for other persons [15]. Patients with psychosis are often considered more difficult to assess in terms of their potential for harm than other patients. In particular, intoxicated patients with psychosis are often secluded by staff to avoid unrest on the ward. Contrary to our expectations, coercive measures were not more frequent among individuals with personality disorders, nor were they more prevalent in the evening or at night (Table 1). The latter finding seems to contradict the hypothesis that seclusions in suicidal patients are merely consequence of understaffing.

The hypothesis that young men might be particularly prone to being secluded was also not confirmed by our results, underscoring the potential to treat this group of acutely suicidal patients without secluding them. Significant differences were found between secluded and nonsecluded patients regarding the circumstances of their admission to the clinic. Patients who had been compulsorily admitted and/or who had been referred by police were more often secluded. Our qualitative data analyses did not reveal any evidence of increased (auto)aggressive behaviour in these patients. It is possible that the circumstances of their admission increased concerns about a higher risk of harm and thus resulted in a more frequent use of coercive measures by the staff.

In Switzerland, approximately 20% of patients are admitted to a psychiatric hospital compulsorily, which corresponds to 1.7 cases per 1000 inhabitants. In the canton of Zurich, the rate is 2.06 cases per 1000 inhabitants and therefore above the Swiss average [30].

The rates of secluded suicidal patients varied considerably between the five wards (13.0% to 55.3%). Different attitudes of the staff towards seclusion or security and fundamentally different team cultures could be reasons for this finding. The only way to avoid coercive measures is to create a general coercive-free attitude that is obligatory for all professionals and to provide the resources necessary to cultivate such an attitude. Another reason for the differences could be the different number of seclusion rooms on each ward (1–2) – if a seclusion room is easily available, its use might be more likely. A detailed investigation of this issue was not possible within the scope of our work and would require further evaluation.

Asylum status and language skills differed significantly between the two groups, with limited language skills and asylum status implying a higher likelihood of being secluded. Only the language barrier was an obstacle to sufficient communication. Qualitative review of medical record entries revealed no evidence of high-risk behaviours. Presumably, staff preferred the safer option of seclusion when faced with an uncertain assessment of a treatment situation. Asylum seekers with refugee experience, however, are a particularly vulnerable patient population and are more likely to exhibit symptoms of posttraumatic stress disorder. Coercive measures can exacerbate clinical symptoms or even provoke posttraumatic stress symptoms in these patients [3, 6]. In this context, training on aspects of transcultural psychiatry could provide an opportunity to improve treatment, particularly with regard to posttraumatic stress disorder. A critical reflection on institutional transference and defence mechanisms may also contribute to counteracting the risk of repeating traumatic incidents within the institution. Developing a general therapeutic attitude among teams on acute care wards may help to maintain a quality of reflection and therapeutic care [26, 31]. In this way, sensitivity towards specific treatment situations might be improved, and a possibly excessive need for protection on the part of the staff might be reduced. The language skills of staff should be utilized, and low-threshold access to translators and cultural mediators should be granted [26]. These measures can help overcome language barriers and thereby reduce coercive measures related to lack of language proficiency and cultural understanding. The provision of an adequate infrastructure can furthermore help to reduce the use of coercion. For instance, so-called Snoezelen rooms, in which patients can adapt the lighting, atmosphere or sounds to their specific needs, might provide an alternative to seclusion rooms. This non-directive therapy where patients control the multisensory environment showed promising results in patients with developmental disabilities and dementia [32]. The seclusion rooms of our hospital had a window and an alarm button to ask for help but otherwise were very sparsely furnished with smooth walls and a mattress, a pillow and a blanket only in order to prevent self-harm. Last but not least, an aggression-free therapeutic approach and a culture of acceptance that is also endorsed and promoted by the hospital management are crucial to reduce the use of coercion. For treatment teams, training on therapeutic principles, intensive care, management of aggression, medical conditions, etc. is required. This should be supported by regular supervision, including a follow-up discussion of difficult situations with an open and appreciative but also self-critical attitude. Following the findings of this study and the recommendations of a recently initiated "commission on coercive measures" in our hospital, we started with inter-professional training sessions to better teach our staff (psychiatrists, psychologists, nurses, etc.) in interventions for patients at risk for coercive measures. In addition, the wards recently intensified the organization of conferences after coercive events where affected patients and the involved staff reflect the event and try to learn of each other for the future. At the patient level, open and empathetic communication involving relatives and respect for the patient's needs are essential. This is best captured by the concluding words "We go to extremes and talk to each other."

### Limitations

The following limitations must be considered when interpreting the study results. Since the data were collected from only one clinic in Switzerland, the generalizability of the findings to other clinics and countries is limited. The results should be reproduced in a larger study with several clinics, ideally within an international context. The qualitative analysis of the digital patient records was performed by trained raters. Documentation of data (e.g., on alternative measures) was, however, not standardized, which made data collection difficult and sometimes not intuitive.

Information on patients' sociodemographic and clinical characteristics was based on routinely collected data. However, acceptable validity of routine clinical diagnoses as defined by the ICD-10 has been demonstrated previously [33].

### Conclusions

To avoid seclusion due to suicidal tendencies, it is necessary to have a general attitude of avoiding coercive measures at all costs. It is also important for qualified staff to be able to deal with challenging sociodemographic characteristics of patients such as foreign-language, which may require translators and intercultural interpreters. Unfortunately, the exact reasons for not providing alternative measures in secluded patient were not apparent in our qualitative survey of the medical records. Further qualitative studies seem warranted to explore why alternative measures are omitted in some cases (e.g. too low a staffing ratio?) and which alternative measures are particularly effective to prevent seclusion in suicidal patients.

## Data Availability

The datasets analysed during the current study are not publicly available due to Swiss data protection laws but are available from the corresponding author on reasonable request.

## Abbreviation

ICD-10: International Statistical Classification of Diseases and Related Health Problems. 10^th^ Revision.
